# A Systematic Review to Examine the Evidence in Developing Social Prescribing Interventions That Apply a Co-Productive, Co-Designed Approach to Improve Well-Being Outcomes in a Community Setting

**DOI:** 10.3390/ijerph18083896

**Published:** 2021-04-08

**Authors:** Gwenlli Thomas, Mary Lynch, Llinos Haf Spencer

**Affiliations:** 1 School of Health Sciences, Bangor University, Bangor LL57 2EF, UK; m.lynch@bangor.ac.uk; 2 Centre for Health Economics and Medicine Evaluation, Bangor University, Bangor LL57 2PZ, UK; l.spencer@bangor.ac.uk

**Keywords:** social prescribing, co-production, co-design, patient-centred care, effectiveness assessment, health, well-being, health equity, social determinants of health, healthy people programs

## Abstract

This systematic review aims to investigate the evidence in applying a co-design, co-productive approach to develop social prescribing interventions. A growing body of evidence suggests that co-production and co-design are methods that can be applied to engage service users as knowledgeable assets who can contribute to developing sustainable health services. Applying the Preferred Reporting Items for Systemic Reviews and Meta-Analyses (PRISMA) guidelines, a systematic literature search was conducted. Peer-reviewed articles were sought using electronic databases, experts and grey literature. The review search concluded with eight observational studies. Quality appraisal methods were influenced by the Grades of Recommendation, Assessment, Development and Evaluation (GRADE) Framework approach. A narrative thematic synthesis of the results was conducted. The evidence suggests that a co-design and co-productive social prescribing can lead to positive well-being outcomes among communities. Barriers and facilitators of co-production and co-design approach were also highlighted within the evidence. The evidence within this review confirms that a co-production and co-design would be an effective approach to engage stakeholders in the development and implementation of a SP intervention within a community setting. The evidence also implies that SP initiatives can be enhanced from the outset, by drawing on stakeholder knowledge to design a service that improves health and well-being outcomes for community members.

## 1. Introduction

Many long-term health issues have their route in socioeconomic and psychological issues that medical interventions cannot always sufficiently surmount [1]. Social Prescribing (SP) provides healthcare professionals with the option of referring patients to various local, non-clinical support groups within their community [2,3]. Groups and organizations receiving referrals may include exercise groups, hobby groups, advice services as well as opportunities to participate in voluntary work and further education [4]. Existing evidence indicates that such interventions can reduce the effects of social determinants of health [5] and avoid the medicalization of social issues [1]. Evidence of such improvements also suggests that SP interventions have the ability to encourage inter-sectoral action which is necessary in tackling the “wicked problem” of health inequalities rooted in the effects of socioeconomic deprivation ([6], p. 1). By connecting individuals with local support groups, SP has been proven effective in reducing social isolation as individuals build new relationships and a social network of support within their communities [5]. SP can therefore lead to improvements in well-being and empower patients to develop resilience to challenging personal situations affecting their health. Consequently, individuals report an increase in self-confidence and self-esteem [7]. Evidence suggests that such emotional improvements can alleviate long-term mental health issues such as anxiety and depression [8].

In addition, patients with long-term physical conditions have also reported becoming self-sufficient in managing their conditions as SP interventions can also connect individuals with groups and organisations that aim to establish healthy lifestyle behaviours [3,8]. Furthermore, evidence of SP interventions leading to healthier lifestyles and self-sufficiency in managing long-term conditions among participants is key given that the World Health Organization’s agenda for sustainable development also includes preventing and treating one third of premature mortality form non-communicable diseases [9]. Such outcomes indicate that SP interventions also could alleviate pressures from overstretched primary care service [7] consequently leading to more sustainable healthy communities and health services.

However, when assessing SP interventions it is imperative to be cognisant of the limited evidence base which mainly consists of small-scale evaluations [10] that are often poorly designed and reported [11]. Previous reviews conclude by pointing to the need for a common SP evaluation framework to overcome such difficulties and facilitate the cross-site comparison of interventions and sharing of results [4,10,11]. Existing evidence indicates that SP is a developing concept and there is a variation in approaches to SP referrals and modes of delivery [4], along with SP providers and end-users [12] This variation further hinders drawing together key findings on SP interventions, in addition to studies not being published and activities not being labelled as SP interventions [13].

Since SP is a person-centred intervention, it was decided to ascertain the effectiveness of applying a co-produced and co-designed approach to its development. The term co-production is generally understood to mean a mutual relationship between service providers, service users and their families and communities [14]. The term encompasses a wide range of service activities including co-design, co-delivery as well as co-assessment of services [15]. Co-design is therefore considered an essential part of full user-professional co-production within the literature although, like other co-productive activity, can also be implemented in isolation [15]. Due to the focus on establishing equality between all stakeholders, studies of co-produced services demonstrates patients treated as knowledgeable assets who can contribute to the design, delivery, and assessment of effective health care services [16]. As a result, the evidence indicates that a co-productive and co-design approach within health challenges the current model of patient health care which is primarily focused on critical illness and views patients as passive users [17]. During co-production sharing of knowledge is democratised as patients’ experiential and implicit knowledge is valued rather than the formal and explicit knowledge based on clinical evidence that can be found in practice guidelines. As a result, patients views are considered equal to those of professionals and consequently co-production descends the traditional power relationship where the clinician is in a position of privilege and the patients is a passive receiver of their expertise [18]. Therefore, co-production and co-design are two approaches that can transform health service into a patient-centred provision.

Previous studies have stated that a co-productive and co-designed approach is necessary in the development of interventions that seek to improve community well-being outcomes. Examples of such interventions include healthy aging programs [19,20] non-medical mental health interventions [21], community-based support for young onset dementia [19] and a mobile health programme to reduce obesity [22]. This statement is based on the perception that each community has unique socioeconomic and environmental features that influence the community’s well-being [20]. As a result, generic interventions will not lead to positive outcomes in every situation and engaging community members in the development of well-being interventions through co-production and co-design makes explicit the main priorities for well-being improvement, resulting in a practical and effective intervention [19,21,22,23]. The evidence indicate that co-production and co-design can also empower communities [22,24] and enable them to have a sense of ownership of an intervention [23] consequently encouraging their participation in the delivered service [20].

As yet, no systematic review has examined the evidence on SP interventions that apply a co-productive and co-designed approach to improve well-being. This review aims to examine evidence of such interventions within community settings. The objective is to review the evidence base to establish current standards in SP that engage communities in co-design and co-production leading to improvements in well-being as well as examine barriers and facilitators to SP intervention development. Community well-being outcomes will also be assessed as an indicator of the SP interventions’ effectiveness.

## 2. Materials and Methods

The protocol for this review was registered on the University of York, Systematic Review database, PROSPERO [25]. The Patient/Problem or Population, Intervention, Comparator, and Outcome (PICO) framework was used to construct the review question which is a mnemonic used in evidence-based practice to frame and answer a clinical or health care related question (see Appendix A) [26]. The framework also influenced the accumulation of search terms. The main keywords were organised into “population,” “intervention,” and “outcomes” groups to ensure that the correct articles were identified. No comparator was included. Search terms included a combination of Medical Subject Heading (MeSh) and non-MeSh words collected by looking at similar reviews search strategy and approaching personal contacts (see Appendix B for a complete list). A Health Sciences specialist Bangor University librarian was consulted to help finalize the search terms and truncate keywords. Search terms were connected with “or” Boolean operators within groups and with “and” Boolean operators between groups. The literature was searched from 2000 to August 2020. Due to limited translation resources the searches were limited to studies published in English.

The following databases were searched: Web of Science; CINAHL; ASSIA; PsycINFO; PubMed incorporating MEDLINE; The Cochrane Library (including the Cochrane Central Register of Controlled Trials). Targeted searching was also conducted on the CRD database. Additional search strategies included hand-searching the key journals within the database search results, targeted searching of grey literature on Google and Google Scholar, and enquiring personal contacts within the field. Appendix C includes a breakdown of the number of records identified on each individual database.

The inclusion criteria were all papers relating to SP interventions that apply a co-productive or co-designed approach to improve well-being outcomes in a community setting. There was no restriction on study type. In this present review well-being is defined as people’s feelings, how they function on a personal and social level and their own overall evaluation of their lives [27]. Communities is defined within this review as a group of people with diverse characteristics but united by social ties, common perspectives and participation in a unified action within a geographical location or setting [28]. The exclusion criteria included studies not related to SP interventions that apply a co-productive, co-design approach to improve well-being outcomes in a community setting.

**Figure 1:**
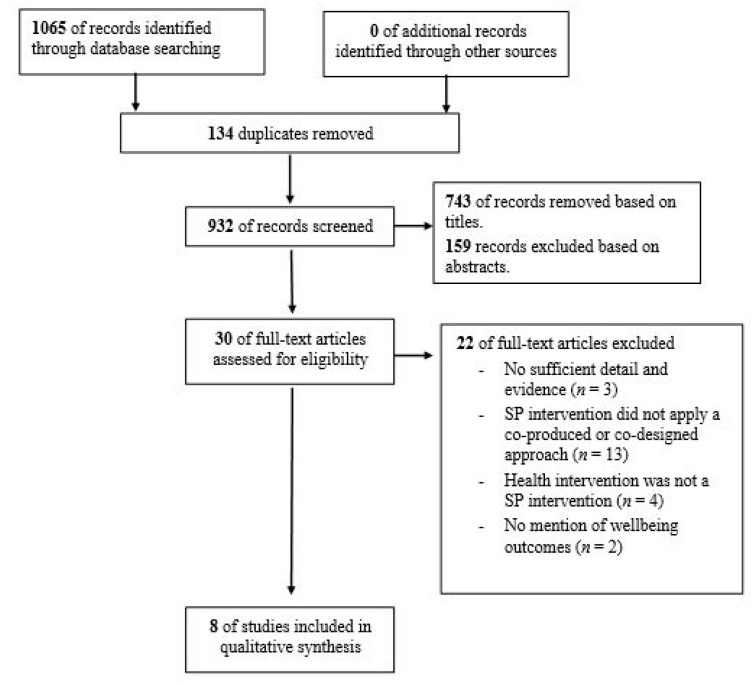
Preferred Reporting Items for Systemic Reviews and Meta-Analyses (PRISMA) [29] flow diagram for search outcomes and screening process.

Articles were initially screened by two reviewers (G.T. and M.L.) for relevance against the eligibility criteria based on their titles. Two reviewers independently assessed the remaining studies by their abstracts and keywords, and all reviews considered relevant were obtained in full. A consensus was reached and documented on all articles meeting the inclusion and exclusion criteria. Disagreements were resolved through a discussion with the third reviewer (L.H.S.). See Figure 1 for a flowchart of the search outcomes and the screening process. Eight articles were identified as relevant and eligible for inclusion. Appendix D contains a list of the 22 full-text articles that were excluded alongside the reasons for their exclusion.

Included studies were subdivided into themes and two reviewers independently extracted data from the final included papers. Data extraction forms were created for the review and piloted on the final included studies. Final data extraction criteria included: Study characteristics; Participants characteristics; intervention content and context; data collection methods and outcomes. All final included papers were critically appraised for methodological quality. Quality appraisal methods were influenced by the GRADE Framework [30]. Following the GRADE framework approach, the quality of each study was initially determined based on the study design and was further assessed according to:

- The clarity of the study’s aims and objectives
- Risk of bias (a scoping level of risk of bias has been determined after considering the risk of confounders, selection bias, allocation bias (if randomized), performance bias, detection bias, attrition bias and measurement bias)
- Indirectness (did the paper state clearly what the population, intervention and outcomes were and did they address the relevant population, interventions, and outcomes for this review question)
- Tests of significance and their results
- Publication bias (were all outcome stated to be measured reported or did the study authors fail to report outcome that showed no (or a negative) effect? Is there any chance of funding bias?)

Following assessment of all the above factors an overarching quality level was determined for each study using GRADE levels. Quality appraisal outcomes are presented in Table 1.

## 3. Results

### 3.1. Overview of Included Studies

The included studies objectives and data collection methods are presented in Table 2.

All studies [31,32,33,34,35,36,37,38] included SP intervention that led to an improvement in well-being outcomes within a community setting. The characteristics of the SP interventions, including the how the co-produced or co-designed approach was applied to each intervention are shown in Table 3.

Two themes emerged among the studies. A proportion of the studies (*n* = 3) studied a co-produced and co-designed approach to the development of a SP intervention to improve well-being within a community setting. These studies considered the dynamics and characteristics of the collaboration between service providers and service users and their communities. The remaining studies (*n* = 5) analysed the community outcomes and perspectives of a SP intervention that applied a co-produced or co-designed approach to improve the community’s well-being. A thematic synthesis [39] of the outcomes under each theme follows.

### 3.2. Theme 1: Co-Produced and/or Co-Designed Approach to SP

All included studies [31,35,38] concerned SP interventions developed in collaboration with service providers, service users and their community. Although co-production is only directly mentioned within the development of SP the intervention in one study [31], co-production and co-design elements can be found in the development of the SP intervention in the two remaining studies.

All studies included within this theme were deemed a low quality (see Table 1). This was on account of being observational studies and the risk of bias assessed in each study, due to the likelihood of selection bias and measurement bias. However, the studies present common sub-themes and offer valuable insight into some of the common challenges and facilitators of co-producing and co-designing a SP intervention to improve well-being outcomes within a community setting.

#### 3.2.1. Realignment

The evidence demonstrates that applying a co-productive and co-designed approach to SP requires a cultural shift. The depth and success of co-production within the evidence varies according to how successful the different co-producers were in bringing the norms and values of different organisations [31,35,38]. This was particularly evident within a study that demonstrated the unsuccessful co-production of a transdisciplinary Social Prescribing intervention, combing art and medicine [31]. The responsibility for co-production was assigned to “boundary spanners” defined within the evidence as individuals within organizations responsible for coordinating various organizational structures and resources in order to organize and govern collaborative ventures [31] (p. 382). The failure of co-production was partly due to the desire of some boundary spanners to dominate while others failed to understand the norms and values of other organizations.

It was also evident that this cultural shift entailed a power shift since equal relationships and mutuality was required between co-producers [35,38]. The evidence demonstrated that effective leadership was necessary to champion the equal relationships and promote collaboration [31,35,38]. Effective leadership was reported to include surrendering autonomy and embracing adaptability on a grassroot level [35,38]. The evidence suggests that such methods ensured that decisions were made for the benefit of the greater community and enabled a sense of ownership of the SP intervention among community members [38].

The evidence indicates that co-production failed where equal relationships were not established. This failure was illustrated by a sense of hierarchy that remained as the traditional model of care prevailed. Health professionals continued to feel most competent and believed that voluntary and community organizations could not adequately address their patient’s needs [31]. It was reported that this sense of “professional preference” towards health professionals also remained due to patients’ expectations [38] (p. 117). The evidence implies that patients can have misperceptions about community and voluntary organisations’ in addition to a reluctance to also seek support from volunteers within their community [38]. When such hierarchy prevailed, the evidence suggest that third sector and voluntary services were approached as additional support rather than complementary to traditional, medical solutions [35,38]. As a result, the evidence indicate that lack of equal relationships prevented the holistic approach to the delivery of positive well-being outcomes.

#### 3.2.2. Sustainability

Attention was also given within the evidence to the sustainability of the collaboration between the different sectors delivering the SP intervention. The evidence also demonstrates that the degree of communication between stakeholders contributed immensely to the long-term sustainability of the co-produced and co-designed SP intervention. Communication was essential to ensure that a relationship was built and maintained between the co-producers. The evidence indicate that it also ensured that each stakeholder felt involved in each stage of the development and subsequent delivery of the intervention [35]. Many facilitators of communication were mentioned within the SR evidence. Co-location or “physical proximity” enabled service providers from different sectors to build close relationships and share information within informal settings [35]. The evidence indicates that these relationships in themselves were also essential in sustaining co-production since health professionals were more likely to refer a patient to a trusted acquaintance [35]. Perhaps the most effective medium of communication emphasized in the evidence was a feedback system. The evidence illustrates that it provided a regular reminder of the existence and benefits of the SP intervention to health professionals consequently encouraging referrals [31,35,38].

In addition to communication, the evidence also suggests that shared resources or systems between the different sectors (e.g., integrated IT system and a single point of contact for referrals) brought convenience and consistency [35,38].

#### 3.2.3. Importance of Evaluation

The evidence highlights the importance of evaluating the intervention from the outset. A lack of evaluation meant that GPs and health professionals were less likely to continue their contribution to the co-production of the intervention in the long term, due to healthcare professionals’ responsibility to prescribe effective and unharmful resolutions [31]. However, the evidence demonstrates that evaluating the intervention was hindered by a lack of a suitable evaluation framework [31]. It was reported that GPs need data presented in a certain way, often using quantitative measures, in order to be persuaded that the SP intervention leads to positive well-being outcomes [31,35]. The task of applying such evaluation frameworks fell on VCS organisations who found the task challenging [35] and preferred qualitative measures [31]. The importance of overcoming such challenges was exemplified within the evidence as failure to sufficiently evaluate one pilot SP intervention contributed to the health sector’s decision not to provide long-term funding for the intervention [31].

#### 3.2.4. Resources

Another observation in each study was that collaboration depended on adequate provision of the necessary resources. Necessary resources included the investment of time to develop the collaborations. The evidence indicates that for SP to work, healthcare professionals should be ready to adapt a more holistic model of health which entails making time to assess patients’ well-being and become acquainted with community resources of support. GPs reported that they did not always have the time to a fully assess patients’ well-being and therefore, could not make referrals [31]. Similarly, it was reported that GPs were detached from the Voluntary and Community Sector (VCS) as they often did not have time to raise their own awareness of the support they could offer patients and develop relationship with the VCS staff [35,38]. The evidence suggest that this was less of an issue where there were pre-existing relationships between healthcare professionals and VCS organisation staff. Such relationships also assisted in establishing mutuality and trust between partners [35,38].

In terms of physical resources, concerns about the lack of consistency within the third sector organisations capacity were reported within the evidence. The VCS organisations were often dependent on short-term funding, which resulted in an “unintended unreliability” [38]. The evidence indicates that GPs were resistant to refer a patient to such uncertain provision of support and were more likely to refer to well-established organisations [31,35,38]. However, it was also acknowledged within the evidence that SP had the potential to increase the numbers of referrals to such organizations, which could, in the long run, strengthen any applications for increased funding [38].

### 3.3. Theme 2: Community Outcomes and Perspectives

Other publications identified in the SR focused on wider community outcomes and/or their perspectives of co-produced co-designed SP interventions within community settings. Five studies were included under this theme which consisted of four case studies [32,33,34,37] and one mixed method survey [36]. All five studies were deemed of low quality on account of being observational studies. A moderate risk of bias was also assessed due to the risk of confounders [36,37], selection bias [33,36,37] and measurement bias [32,33,34] As within the previous theme, there is consistency in terms of the valuable outcomes and perspectives reported in each study which increases the credibility of the results.

An increase in confidence was a common well-being outcome reported within the studies. This was mainly as a result of a reduction social isolation as the SP intervention motivated participants to join social groups and build a social network of support [32,34,36]. The evidence suggested that applying a co-productive and co-designed approach to SP gave individuals a sense of control that also increased their self-confidence and often led to a positive mood [34]. Such improvements were particularly appreciated by individuals suffering from isolating mental health issues [34]. Individuals reported that they had developed strategies to deal with their situation and as a result gained the confidence and self-esteem they desperately needed [34].

As well as giving individuals this sense of control, being able to co-produce or co-design with the SP intervention provider also meant that participants felt the staff were approachable, which encouraged their participation [36]. The evidence demonstrates that participants greatly appreciated being listened to as they co-designed their social prescription with a support worker. This was reported as a positive change from being treated as passive users by healthcare professionals [33,34].

The evidence also suggests that the reciprocal relationships established between service users and service providers were particularly beneficial in creating positive well-being outcomes. Participants reported that being able to help others within a similar situation was rewarding and empowering as it led them to realize the strengths and weakness in themselves and others [33,34]. There was also evidence that sharing experiences and coping mechanisms motivated newer service users and gave them the hope that they could achieve the same positive well-being outcomes [37].

The studies also provided insight into the possible obstacles that prevented service users from participating in the SP interventions. The reported obstacles were mainly due to the individual’s personal situations. One of the most apparent obstacles within the evidence was lack of transport options [32,33]. In addition, many felt a lack of confidence due to social isolation, felt restricted due to depression [33] and felt nervous about joining new groups due to a negative previous experience [32]. The cost of the service as also a barrier reported within one study [36]. However, Blickem et al. [32] study highlights that co-designing a SP intervention gives service providers and users an opportunity to discuss concerns and design the intervention in a way that could overcome any obstacles from the outset.

The evidence also demonstrated the power of creating opportunities for SP practitioners to reflect on participants outcomes and perspectives. Reflecting on such findings through an action learning framework was shown to enable practitioners to explore participants to achieve positive well-being outcomes and the implications for their own practice [33]. Establishing such “cycles of questioning, planning, experimentation and reflection” was considered good practice to develop efficient and effective interventions and strengthen collaboration across disciplines and organizations [33] (p. 70).

## 4. Discussion

This systematic review set out to examine the evidence in developing SP interventions that apply a co-productive, co-designed approach to improve well-being outcomes in a community setting. The evidence demonstrates that co-production and co-design can be an effective way of engaging stakeholders in the development of a SP intervention to improve well-being in a community setting. Consistent with other studies of co-produced and co-designed community well-being interventions [20] it was reported that patients value the patient-centred approach that entails being approached as individuals, not passive users. Similar to previous studies of a co-productive, co-designed approach within health [17,40,41] the evidence demonstrates the establishment of a mutual relationship between service providers and service users as a transformative process. Existing evidence of co-production and co-design in health indicate that the idea of creating equal relationships and stepping away from the traditional model of health is unrealistic [42]. However, the evidence within the present study illustrates that it is possible and essential within the co-production of a SP intervention as failure to establish mutuality was reported to have created a sense of hierarchy and distrust [31].

The evidence indicates that positive well-being outcomes were achieved as a result of such an approach. Well-being outcomes across the included studies were reported to have been an increase in confidence, empowerment, and self-sufficiency as well as reduction in social isolation. Positive well-being outcomes were reported among individuals with long term conditions [32,33,36], mental health problems [34] and, a co-produced SP intervention also led to feelings of “connectedness” among individuals living with early onset dementia [31] (p. 385). They were also evident among larger, deprived communities suffering from health inequalities [35,37].

However, the SR evidence also indicates clearly that there are facilitators and barriers that can influence the success of the co-production and co-design of a SP intervention within community settings. Effective leadership was reported essential in advocating the necessary mutuality between co-producers [35,38] a finding that mirrors those of other studies of the collaborative development of health programs in community settings [43]. Effective communication is also essential in sustaining and enhancing the personal relationships between stakeholders as well as implementing a suitable evaluation framework to ascertain the effectiveness of the SP intervention from health professionals’ perspective [31,35,38]. Previous studies of co-production have stated that it is effective practice to extend the approach to co-assessment following the co-delivery of a service [15,44]. The need to find an evaluation framework suitable for all stakeholders arguably strengthens the case for following such procedures. A study of evaluation methods for arts, health and well-being projects found that the co-production of evaluation methods is time consuming but can ensure that the evaluation framework is fully embedded in service delivery and draws upon the knowledge and skills of all stakeholders, ensuring their buy-in from the outset [45]. In accordance with other studies that have assessed collaboration within community care [43,46] results of the current review indicates that the sense of trust between health professionals and SP providers owing to an effective evaluation was also crucial to the delivery of the SP intervention.

This review also found that a context with adequate resources is also vital to the sustainability of co-produced and co-designed SP interventions. Similar to other studies of co-produced and co-designed health interventions [42] this review touched on the importance of ensuring that health professionals are prepared to devote time to co-production and co-design [31,35] However, sufficient financial resources were the main resources required according to many of the authors [31,35,38]. This finding is supported by studies that highlight that the design of a resilient intervention requires assessing the available resources to determine what is financially feasible [46] and establishing realistic goals and objectives to avoid loss of motivation among stakeholders [43].

## 5. Study Strengths and Limitations

A key strength of the present study was the explicit inclusion and exclusion criteria that were applied to discover relevant studies that could achieve this study’s aim and objectives. In addition, a second and third reviewer were consulted during all stages of the review process to increase the robustness of the review and reduce the risk of bias. However, despite attempts to avoid publication bias, the current review only searched for studies published in English due to limited translating resources. Therefore, it must be acknowledged that the search strategy may not be comprehensive. The articles found in the review are mostly qualitative, however the quality assessment criteria were influenced by tools designed to evaluate quantitative studies. It is therefore recognized that this aspect could also produce a bias in the interpretation of the results.

As previously discussed, all included studies were of a low-quality standard. In addition to the previously stated limitations, the exact number of participants within some studies is unknown [37] as well as the duration of the SP intervention within most of the studies affecting the reliability of the evidence. Similarly, while all studies met the inclusion criteria meaning that each study’s population was a community, the demographics varied among the studies. In addition, various data collecting methods were used within each study. Both these factors affect the ability to generalize the findings. However, as already mentioned within this review, common subthemes and valuable outcomes were found among the studies, increasing credibility.

## 6. Implications for Future Research and Action

This systematic review has demonstrated that taking a co-designed, co-produced designed approach to SP intervention development, implementation and evaluation has the potential to positively engage with stakeholders and encourages buy-in and utilisation among end users and sense of ownership for the intervention. Stakeholder engagements also leads to a SP intervention that is effective and efficient in meeting the health needs as well as improving health outcomes of end-users. The evidence presented within the current review therefore suggests that a co-designed and co-produced also contributes to the development of sustainable healthy communities as it leads to interventions that are tailored to community needs and available community resources. This finding reinforces previous studies that have highlighted that engaging providers and end-users in the development of SP interventions is key to sustainable SP interventions [47]. As a result, this review suggests that applying a co-designed, co-produced approach to the design of SP interventions in future would be effective practice, particularly within a community setting.

However, this systematic review also indicates that the current evidence base of co-designed, co-produced SP interventions is limited and consisting of low-quality studies. Future recommendations for SP research include high quality studies that include all stakeholders to further confirm what makes such an approach effective within the development of SP interventions as well as limiting its success. The evidence also demonstrates a lack of effective evaluation of co-produced and co-designed SP interventions. Consequently, to improve the quality standards for research and reporting future co-design and co-production of SP interventions should also build evaluation in from the inception phase onwards to the implementation. As the field of SP is an emerging area, there are many examples of frameworks that can be used to facilitate such steps such as a Realist Evaluation [48] and Social Return on Investment [49]. Such approaches will demonstrate the effectiveness and impact of SP interventions in meeting health needs and improved health outcomes ensuring health equity.

## 7. Conclusions

The evidence strongly suggests that a co-production and co-design would be an effective approach to engage stakeholders in the development and implementation of a SP intervention within a community setting. The results of this review also indicate that SP initiatives can be enhanced from the outset, by drawing on stakeholder knowledge to design a service that improves community members health and well-being outcomes. Taken together, the facilitators and barriers of co-production and co-design highlighted within the evidence suggests how to efficiently implement such an approach to the development of a SP intervention within a community. When a co-production and co-design is successfully applied, the evidence illustrates that it can improve well-being outcomes, and communities feel empowered by this patient-centred approach. However, caution must be applied since this review consists of only a small number of low-quality studies. Therefore, SP interventions that apply a co-productive, co-designed approach to improving well-being outcomes in a community setting require more, high quality research to further investigate which mechanisms of such an approach lead to better well-being outcomes for communities.

## Figures and Tables

**Table 1:** Quality Assessment Results.

Study Author (Year) [Reference]	Study Design Certainty	Study Aim and Objective Clearly Stated	Risk of Bias	Indirectness	Publication Bias	Test of Significance	Overall Quality
Baker and Irving (2016) [31]	Low	No	Moderate risk	No serious indirectness	No serious risk	No information	Low
Blickem et al. (2013) [32]	Low	Yes	Moderate risk	No serious indirectness	No serious risk	No information	Low
Chesterman and Bray (2018) [33]	Low	No	Moderate risk	No serious indirectness	No serious risk	No information	Low
Hassan et al. (2020) [34]	Low	Yes	Moderate Risk	No serious indirectness	No serious risk	No information	Low
Southby and Gamsu (2018) [35]	Low	Yes	Moderate risk	No serious indirectness	No serious risk	No information	Low
Strachan, Wright and Hancock (2007) [36]	Low	Yes	Moderate risk	No serious indirectness	Moderate risk	No information	Low
Swift (2017) [37]	Low	Yes	Moderate risk	No serious indirectness	Moderate risk	High significance	Low
Whitelaw et al. (2017) [38]	Low	Yes	Moderate risk	No serious indirectness	No serious risk	No information	Low

**Table 2:** Study Characteristics.

Study Author (Year) [Reference]	Study Design and Methods	Objectives	Participants	Social Context
Baker and Irving (2016) [39]	Case Study consisting of review of project documentation; semi-structured interviews; focus groups; observations of Steering Group meetings.	To address the gap in the literature regarding the role of boundary-spanners in supporting or enabling the co-production of an arts-based, pilot SP scheme.	People living with early-onset dementia at risk of depression and their family members, project steering group, GPs and other primary care staff, Community Art Organisation, sheltered accommodation wardens.	Various community venues and sheltered accommodation in North East England.
Blickem et al. (2013) [32]	Qualitative Study using focus group and interviews.	To combine insights from service users with long-term conditions to develop a SP intervention to promote engagement and improve access to health-relevant resources.	Individuals living with long term conditions attending health and well-being support groups.	Greater Manchester, England.
Chesterman and Bray (2018) [33]	Action Research, Appreciative Inquiry and Action Learning	To complement the formal evaluation of schemes established by the Crawley SP Partnership with targeted Action Research. Co-researchers were members of the Crawley SP Partnership.	Co-researchers were members of the Crawley SP Partnership. The interviewees suffered from long term conditions and participated in well-being activities.	Various community venues in Crawley, England
Hassan et al. (2020) [34]	Qualitative study using focus groups	To explore elements that contribute toward enhancing a SP model addressing the social determinants of mental health.	Individuals from Mersey Care NHS Foundation Trust who had accessed The Life Rooms between September 2017 and April 2018.	Life Rooms, Liverpool and Sefton, England—one of the most disadvantaged areas in the country.
Southby and Gamsu (2018) [35]	Case Study using semi-structured interviews and focus group	To add to the knowledge base around collaborative practice between GPs and Voluntary and Community Sector (VCS) organisations by examining four SP schemes.	GPs and VCS organisations involved in four SP schemes.	Communities in Sheffield experiencing significant health inequalities.
Strachan, Wright, and Hancock (2007) [36]	Survey using open and closed questionnaire.	To examine the extent to which SP participants have experienced improvements in their health and well-being.	Tailor Made Leisure Package applicants over 16 years of age.	Healthy Living Centre, Scotland.
Swift (2017) [37]	Case Study. Well-being outcomes were measured using Subjective well-being (SWEMWBS). The report also refers to qualitative data collected to determine the broader impact of the intervention on patient lives.	To discuss a co-designed community-centred approach to health.	Patients at all 17 GP practices in Halton who had been referred to the SP interventions.	Community venues in Halton, England, an area with high levels of deprivation and signs of health inequalities.
Whitelaw et al. (2017) [38]	Case study using 1:1 semi-structured interviews	To conduct a process-based evaluation of the inception and early implementation of a SP initiative.	The project steering group; staff of two primary care organisations and the varied community resources associated with the project.	Two GP practices in Scotland. The communities were rural in nature with low population density and relatively high levels of isolation.

**Table 3:** SP intervention characteristics and outcomes.

Study Author (Year) [Reference]	Name, Location and Description of Intervention	Co-Produced or/and Co-Designed Approach
Baker and Irving (2016) [31]	Arts-based SP provided from various community venues in North East England to combat problems of isolation and loneliness among and improve the well-being of older people with early onset dementia and depression.	Developed through a collaboration between a Primary Care Trust and Community Arts Organisation.
Blickem et al. (2013) [32]	An online SP referral tool based on community support providers in Greater Manchester, England for people with long term conditions. Intervention was designed to provide well-being, health education, practical support and help with diet and exercise.	The intervention was developed in collaboration with service users. Noralization Process Theory guided the development in a way in which gradual changes were implemented on the bases of feedback at different stages from the patient.
Chesterman and Bray (2018) [33]	Well-being promoting activities provided by voluntary sector organizations in various community venues in Crawley, England.	SP practitioners were recruited as co-researchers to conduct appreciative inquiry interviews with citizens participating in SP activities. Co-researchers analysed interview data with other SP practitioners to decide on further action and subsequently implemented positive change to the SP intervention.
Hassan et al. (2020) [34]	SP provided from The Life Rooms in Liverpool and Sefton, England. SP intervention involves learning opportunities or social support. There are also advice services on housing, debt, employment, or well-being support. Employment and enterprise volunteering support is also available.	Each social prescription is co-produced with service users, carers, partner organisations and staff.
Southby and Gamsu (2018) [35]	Four SP schemes delivered in GP surgeries and VCS organizations centres aimed at improving well-being.	All SP interventions had been developed and were delivered through a collaboration between GPs and VCS organization. The depth of collaboration varied between each case.
Strachan, Wright, and Hancock (2007) [36]	Tailor Made Leisure Package (TMLP) is a SP intervention delivered from the Healthy Living Centre, Scotland. The intervention was developed to encourage disadvantaged groups to embark on an individual program of exercise and relaxation.	The TMLP is a SP co-designed with the service users to meet individual needs and capability.
Swift (2017) [37]	A community-centred approach delivered from community venues in Halton, England to respond more appropriately to social determinants of health. The approach includes a community-navigation scheme, a SP intervention and a social action element that involves recruiting patients who make use of the SP service to co-facilitate sessions with tutors.	The SP intervention was developed through a Theory of Change that was co-designed with stakeholders with a key emphasis on empowering patients. GPs were consulted before launching the intervention to seek their buy-in and establish a referral process. In addition, the SP is co-designed with the service users, and a co-production approach can also be seen within the Social Action element.
Whitelaw et al. (2017) [38]	A link worker working within two GP practices in rural Scotland assesses patients’ health and well-being needs and refers patients to available community resources.	The project was co-developed by a multi-sector Steering Group.

## Data Availability

No new data were created or analyzed in this study. Data sharing is not applicable to this article.

## Glossary

ASSIA	Applied Social Sciences Index and Abstracts.
CINAHL	Clinical Index for Nursing and Allied Health Literature.
CRD	Centre for Reviews and Dissemination.
GP	General Practitioner.
GRADE	Grades of Recommendation, Assessment, Development and Evaluation.
IT	Information Technology.
MEDLINE	A digital database for journal articles in the life sciences with a concentration on bio-medicine.
MESH	Medical Subject Heading.
PICO	Patient/Problem or Population, Intervention, Comparator and Outcome(s).
PRISMA	Preferred Reporting Items for Systemic Reviews and Meta-Analyses.
PROSPERO	International database of prospectively registered systematic reviews where there is a health related outcome.
PsycINFO	A digital index for the social sciences including Psychological Abstracts.
PubMed	A digital resource supporting the search and retrieval of biomedical and life sciences literature.
SP	Social Prescribing.
SWEMWBS	Short Warwick-Edinburgh Mental Wellbeing Scale.
TMLP	Tailor-Made Leisure Programme.
VCS	Voluntary and Community Sector.

## Appendix A

**Table A1:** Patient/Problem or Population, Intervention, Compartor and Outcome(S) (PICO) framework for mixed methods search strategy.

Population	Intervention	(Comparison)	Outcomes
Any community	Social Prescribing interventions	(No comparison was included)	Improvement in community well-being outcomes

## Appendix B

**Table A2:** Search terms for mixed methods search strategy.

Communities	Social Prescribing	Co-Production and Co-Design	Well-Being
communit *	“social prescri” *	co-design *	well-being NEAR/3 improve *
neighbourhood	non-medical NEAR/3 referral *	codesign *	wellbeing NEAR/3 improve *
society	non-clinical NEAR/3 referral *	co-produc *	“community resilience”
resident *	“non-medical intervention”	coproduc *	“community sustainability”
patient *	“non-clinical intervention”	participat *	“community development”
“service user” *	“community-based intervention” *	collaborat *	“social inclusion”
stakeholder *	wellbeing program *	engagement	“health benefit” *
people	well-being program *	involvement	“mental health benefit” *
	“link worker” *	“jointly produced”	“physical benefit” *
	“community navigator” *	“jointly designed”	“quality of life”
	health facilitator	user-led	
	“social intervention”	co-creat *	
	social NEAR/3 referral	participatory design	
		action research	
		participatory research	
		design *	
		produce *	

Please note that all asterisks (*) were included in the search strategy and used to truncate keywords.

## Appendix C

**Table A3:** Number of records identified in each database.

	Web of Science	PubMed	CINAHL	PsychInfo	ASSIA	Cochrane	CRD Database	Total
Initial number of records	240	136	564	19	73	33	0	1065
Number after removing duplicates	153	132	531	14	70	31	0	931

## Appendix D

**Table A4:** List of full text-articles excluded and reasons for exclusion.

Full Paper Reference	Reason for Exclusion
Elston, J. et al. Does a social prescribing ‘holistic’ link-worker for older people with complex, multimorbidity improve well-being and frailty and reduce health and social care use and costs? A 12-month before-and-after evaluation. *Prim. Health Care Res. Dev*. 2019, 20, doi:10.1017/S1463423619000598.	Excluded due to limited discussion on co-design of SP intervention.
Soraghan, C. J.; Boyle, G.; Dominiguez-Villoria, J. F.; Robinson, D. Challenges of implementing a social prescription service in the clinic: Social prescribing in the LAMP project. *Int. Symp. Technol. Soc. Proc.* 2016, March, 1–6, doi:10.1109/ISTAS.2015.7439434.	Excluded as the SP didn’t apply a co-produced/co-design approach
Moffat, S.; Steer, M.; Lawson, S.; Penn, L.; O’Brien, N. Link Worker social prescribing to improve health and well-being for people with long-term conditions: qualitative study of service user perceptions. *BMJ Open.* 2017, 7, doi:10.1136/bmjopen-2016-015203.	Excluded as the SP didn’t apply a co-produced/co-design approach
Mulligan, K.; Bhatti, S.; Rayner, J.; Hsiung, S. Reply to: Looking Before We Leap: Building the Evidence for Social Prescribing for Lonely Older Adults. *J. Am. Geriatr. Soc.* 2020, 68, 434–435, doi:10.1111/jgs.16254.	Excluded as there is no sufficient detail and evidence.
Wildman, J. M.; Moffat, S.; Steer, M.; Laing, K.; Penn, L.; O’Brien, N. Service-users’ perspectives of link worker social prescribing: a qualitative follow-up study. *BMC Public Health.* 2009, 19, doi:10.1186/s12889-018-6349-x.	No mention of a co-produced nor co-designed approach.
Aggar, C.; Thomas, T.; Gordon, C.; Bloomfield, J.; Baker, J. Social Prescribing for Individuals Living with Mental Illness in an Australian Community Setting: A Pilot Study. *Community Ment. Health J*. 2020, 57, 189–195, doi:10.1007/s10597-020-00631-6.	Since all other keywords were mentioned full paper was read to confirm that there was no mention of co-production/co-design.
Simpson, S.; Smith, S.; Furling, M.; Ireland, J.; Giebel, C. Supporting access to activities to enhance well-being and reduce social isolation in people living with motor neurone disease. *Health Soc. Care Community.* 2020, 28, 2282–2289 doi:10.1111/hsc.13049.	No mention of a co-produced nor co-designed approach.
de Villers, C. Elevate 2018: The Arena of Physical activity, health and performance. Global and local community collaborations to promote and improve physical activity. *Aqualines J. Hydrotherapy Assoc. Chart. Physiother.* 2018, 30, 6–9.	Full text read as there was no abstract. No mention of a co-produced nor co-designed approach.
Wildman, J. M.; Valtrota, N. Moffat, S. Hanratty, B. What works here doesn’t work there’: The significance of local context for a sustainable and replicable asset-based community intervention aimed at promoting social interaction in later life. *Heal. Soc. Care Community.* 2019, 27, 1102–1110, doi:10.1111/hsc.12735.	Health intervention was not a SP intervention.
Katiforis, R. Reducing Harm in the Community. *Aust. Nursing. J. 2007*, 15. [no DOI address found]	Full text read as there was no abstract. Not a co-designed/co-produced SP intervention.
Mechen, C. The collaborative, community approach of the Leg Club model. *J. Community. Health.* 2015, 31, 12–14. [no DOI address found]	Full text read as there was no abstract. Not a co-designed/co-produced SP intervention.
Howarth, M.; Griffiths, A.; Silva, A.; Green, R. Social Prescribing: a ‘natural’ community-based solution. *Br. J. Community Nurs*. 2020, 25, 294–298, doi:10.12968/bjcn.2020.25.6.294	No mention of co-design nor co-production
Mossabir, R.; Morris, R.; Kennedy, A.; Blickem, C.; Rogers, A. A scoping review to understand the effectiveness of linking schemes from healthcare providers to community resources to improve the health and well-being of people with long-term conditions. *Health Soc. Care Community.* 2015, 23, 467–484, doi:10.1111/hsc.12176.	Review of social interventions, including social prescriptions, leading to wellbeing improvements within a community setting but not co-produced/co-designed.
Morton, L.; Ferguson, M.; Baty, F. Improving wellbeing and self-efficacy by social prescription. *Public Health*. 2015, 129, 286–289, doi:/10.1016/j.puhe.2014.12.011.	Full paper was read to confirm that there was no mention of co-production/co-design.
Husk, K.; Blockley, K., Lovell, R.; Bethel, A.; Lang, I.; Byng, R.; Garside, R. What approaches to social prescribing work, for whom, and in what circumstances? A realist review. *Health Soc. Care Community*. 2020, 28, 309–324, doi:10.1111/hsc.12839.	No mention that co-production and co-design contribute to successful referrals.
Chatterjee H.; Polley, M.; Clayton, G. Social prescribing: community-based referral in public health. *Perspect. Public Health*. 2018, 138, 18–19, doi:10.1177/1757913917736661.	A short review of SP evaluations. There is brief mention of co-design within the definition of SP but the paper does not refer to any co-designed/co-produced SP interventions.
Skivington, K.; Smith, M.; Chng, N.R.; Mackenzie, M.; Wyke, S., Mercer, S. W. Delivering a primary care-based social prescribing initiative: A qualitative study of the benefits and challenges. *Br. J. Gen. Pract*. 2018, 68, 487–494, doi:10.3399/bjgp18X696617.	Excluded as there was no mention of health and well- being outcomes
Gellataly, J.; Bee, P.; Gega, L.; Bower, P.; Hunter, D.; Stewart, P.; Stanley, N.; Calam, R.; Holt, K.; Wolpert, M.; Douglas, S.; Green, J.; Kolade, A.; Callender, C.; Abel, K A. A community-based intervention (Young SMILES) to improve the health- related quality of life of children and young people of parents with serious mental illness: randomised feasibility protocol. *Trials.* 2018, 19, doi:10.1186/s13063-018-2935-6.	The community-based intervention was co-developed with stakeholders, but it is not a Social Prescribing model.
Mercer, S. W.; Fitzpatrick, B.; Grant, L.; Chng, N. R.; McConnacbie, A.; Bakhshi, A., James-Rae, G.; O’Donell, C. A.; Wyke, S. Effectiveness of Community-Links Practitioners in Areas of High Socioeconomic Deprivation. *Ann. Fam. Med.* 2019, 17, 518–525, doi:10.1370/afm.2429.	No mention of co-production nor co-design.
Leerlooijer, J. N.; Gerjo, K.; Weyusya, J.; Arjan, E R. B.; Ruiter, R. A. C.; Rijsdijk, L. E.; Nshakira, N.; Bartholomewn, L. K. Applying Intervention Mapping to develop a community-based intervention aimed at improved psychological and social well-being of unmarried teenage mothers in Uganda. *Health Educ. Res.* 2014, 29, 598–610, doi:10.1093/her/cyu020.	Full text was read in order to confirm that the community-based intervention did not resemble a SP intervention.
Ho, H C. Y.; Mui, M. W.; Wan, A.; Yew, C. W. Lam, T. H. Happy Family Kitchen Movement: A Cluster Randomized Controlled Trial of a Community-Based Family Holistic Health Intervention in Hong Kong. *J. Happiness Stud*. 2020, 21, 15–36, doi:10.1007/s10902-018-00071-w.	Full text was read to confirm that the community-based intervention did not resemble a SP intervention.
Blignaut, I.; Haswell, M.; Pulver, L. J. The value of partnerships: Lessons from a multi-site evaluation of a national social and emotional wellbeing program for Indigenous youth. *Aust. N. Z. J. Public Health.* 2016, 40, 53–58, doi:10.1111/1753-6405.12403.	Full text was read to confirm that the community-based intervention did not resemble a SP intervention
